# Impact of veriflex and ICL on corneal biomechanics and endothelial cell density

**DOI:** 10.1038/s41598-025-14330-3

**Published:** 2025-08-27

**Authors:** Passant Sayed Saif, Mohamed Gaber Okasha, Hatem Mahmoud, Asmaa Samir, Mohamed Abdelmonagy Ibrahim

**Affiliations:** 1 Department of Ophthalmology, Misr University for Science and Technology, Giza, Egypt; 2 Department of Ophthalmology, Faculty of Medicine, Al-Azhar University for Boys, Cairo, Egypt; 3 Department of Ophthalmology, Beni-Suef University, Beni Suef, Egypt; 4 Department of Ophthalmology, Fayoum University, Fayoum, Egypt

**Keywords:** Phakic IOLs, ICL, Veriflex, Myopia, Corvis ST, Endothelial cell density, Corneal diseases, Refractive errors

## Abstract

To evaluate endothelial cell density (ECD) and corneal biomechanical changes following implantation of Veriflex and Implantable Collamer Lens (ICL) phakic intraocular lenses (pIOLs) over a 12-month period. Sixty **patients (117 eyes)** were included in this prospective comparative study. **58 eyes** underwent implantation with the Veriflex lens (Group I), while **59 eyes** received the ICL (Group II). Preoperative and postoperative ECD and corneal biomechanical values were assessed using specular microscopy and Corvis St at baseline, 6 months, and 1 year. Endothelial cell loss percentages and corneal biomechanical changes were calculated and compared between the 2 groups. Preoperatively, the mean ECD was **2683.4 ± 340.6 cells/mm**^**2**^ in Group I and **2732 ± 322.8 cells/mm**^**2**^ in Group II (***P* = 0.718**). At 1 year, Group I exhibited a significantly greater reduction in ECD compared to Group II (*P* = 0.021), indicating higher endothelial cell loss with Veriflex. The mean endothelial cell loss at 12 months was **5.4% (145.7 ± 62.8 cells/mm**^**2**^**) in Group I** compared to **1.53% (42 ± 17.3 cells/mm**^**2**^**) in Group II** (***P* < 0.001**). The **Veriflex group** showed greater corneal biomechanical changes, with a **deformation amplitude (DA)** increase to 1.15 ± 0.11 mm and a **highest concavity time (HCT)** of 17.2 ± 1.1 ms, while the **ICL group** demonstrated more stable biomechanics, with a DA of 1.09 ± 0.09 mm and an HCT of 16.6 ± 1.0 ms at one year postoperatively. Both Veriflex and ICL pIOLs resulted in a decrease in ECD over one year, with Veriflex showing a significantly higher rate of endothelial cell loss. These findings suggest that ICL may be a safer option for long-term endothelial preservation. Long-term studies are required to assess the continued impact of both lenses on corneal endothelial health.

## Introduction

Myopia is a prevalent global refractive disorder with increasing incidence, particularly in urban populations, and presents a substantial public health challenge^1^. Moderate to high myopia, defined as a spherical equivalent(SE) refractive error of −6.00 diopters (D), or greater, is associated with an elevated risk of sight-threatening complications, including retinal detachment (RD), myopic macular degeneration, and glaucoma^2,3^. While spectacles and contact lenses (CL) provide effective optical correction, surgical interventions such as LASIK and phakic intraocular lenses (pIOLs) offer long-term refractive stability and precision^2,3^.

Phakic intraocular lenses (pIOLs) have emerged as a compelling alternative for patients with moderate to high myopia and keratoconus, particularly those contraindicated for corneal refractive surgery due to inadequate corneal thickness or extreme refractive errors. Unlike corneal ablative procedures, pIOLs preserve the crystalline lens and accommodate it, ensuring superior optical performance and potential reversibility^3,4^. Among the widely utilized pIOLs, the foldable iris-fixated anterior chamber lens (Veriflex, Advanced Medical Optics (AMO), based in Santa Ana, California, USA) and posterior chamber Implantable Collamer Lens (*EVO ICL V4c* (STAAR Surgical, Monrovia, CA, USA).) are prominent options. Although both lenses achieve effective refractive correction, their distinct anatomical placement influences their safety profiles, particularly in relation to corneal ECD^5–7^.

ECD is a critical determinant of corneal health because the endothelium is responsible for maintaining corneal transparency and stromal hydration. While a natural decline in ECD occurs with age, additional insults from surgical trauma, intraocular inflammation, or mechanical contact with pIOLs can accelerate endothelial cell loss. Excessive endothelial cell density (ECD) depletion may lead to corneal decompensation, potentially necessitating keratoplasty in severe case^8,9^. Consequently, evaluation of the impact of different pIOLs on endothelial cell preservation is imperative to ensure optimal surgical outcomes and long-term ocular safety.

The Veriflex lens, an iris-fixated anterior chamber pIOL, was anchored to the mid-peripheral iris through enclavation. Despite its stable fixation, concerns persist regarding its potential mechanical interaction with the corneal endothelium, which may contribute to progressive endothelial cell attrition^10^. Conversely, the ICL is a posterior chamber lens that resides between the iris and the crystalline lens, thereby minimizing direct endothelial contact and posing a theoretical risk of cataractogenesis. However, advancements in ICL design such as the incorporation of a central port to enhance aqueous humor dynamics have mitigated these concerns^4,10^.

The **Corvis ST** (Scheimpflug Technology) is a non-contact tonometer that provides a comprehensive assessment of corneal biomechanics by measuring the cornea’s dynamic response to an air puff. Utilizing high-speed Scheimpflug imaging, Corvis ST captures real-time deformation of the cornea, offering detailed parameters such as **deformation amplitude (DA)**, **applanation length (AL)**, **applanation velocity (AV)**, **highest concavity time (HCT)**, and **Corvis Biomechanical Index (CBI)**. These metrics are critical for evaluating corneal stiffness, elasticity, and overall biomechanical stability, making Corvis ST an invaluable tool for diagnosing and managing corneal ectatic disorders, screening candidates for refractive surgery, and monitoring postoperative outcomes. By providing insights into the cornea’s biomechanical properties, the Corvis ST enhances our understanding of corneal behavior under stress, aiding in the early detection of conditions like keratoconus and optimizing patient care in refractive and therapeutic interventions^11^.

Understanding these biomechanical properties is crucial for assessing the safety and effectiveness of different pIOLs, which leads us to the **aim of this study** to conduct a comparative analysis of ECD changes and corneal biomechanics following implantation of the Veriflex and ICL lenses in patients with moderate to high myopia over a one year follow-up period. By assessing endothelial cell loss and its clinical significance, this study sought to provide robust evidence to guide the selection of the most appropriate pIOL for myopic correction in clinical practice.

## Patients and methods

This study was conducted in accordance with the ethical principles outlined in the Declaration of Helsinki. Informed written consent was obtained from the patients for both participation and publication of clinical data, imaging, and outcomes. The study was approved by the Research Ethics Committee of Beni-Suef University (approval #: FMBSUREC/05,012,025/Saif). Patients were recruited from the outpatient clinics of Fayoum, Beni-Suef , Azhar and Misr Universities Hospitals. Surgeries were done by two surgeons (group I :MGO, Group II : MAM) while the imaging was done by (PSS). The study included 117 eyes of 60 patients with moderate-to-high myopia divided into two groups:

- Group I (Veriflex): 58 eyes of 30 patients implanted with a foldable iris-fixated anterior chamber pIOL .
- Group II (ICL): 59 eyes of 30 patients implanted with posterior chamber pIOL.

Inclusion Criteria:

- Moderate to high myopia
- Age between 21 and 45 years
- Stable refraction for at least one year

*Exclusion Criteria:*

- Abnormal cornea (e.g., keratoconus, endothelial dystrophy)
- Endothelial cell count < 2600 cells/mm^2^
- Anterior segment pathology (e.g., cataract, pseudoexfoliation, severe iris atrophy)
- Abnormal pupil (> 6.5 mm in mesopic light)
- History or signs of iritis or uveitis
- Glaucoma or family history of glaucoma
- Anterior chamber depth < 3.00 mm
- White-to-white distance < 11 mm (ICL cases)

*Preoperative Assessment*

- Full ophthalmic examination including uncorrected visual Acuity (UCVA) and best-corrected visual acuity(BCVA)
- Manifest refraction
- Slit lamp examination
- Intraocular pressure (IOP) measurement using Goldmann applanation tonometer
- Fundus examination
- Keratometry and anterior chamber depth (ACD) analysis using the Oculus Pentacam HR
- Endothelial cell count measurement using Topcon EM-3000
- Corneal Biomechanics using Corvis ST

*Operative Techniques* Standard procedures for Veriflex^10^ and ICL implantation^3^ were followed, including pupil dilation, incision creation, pIOL loading, insertion, fixation, and postoperative management.

*For Veriflex group* The Veriflex lens (Artisan/Veriflex iris-claw type, AMO, Netherlands) was implanted in the anterior chamber and fixated to the mid-peripheral iris using enclavation technique.

*For the ICL group*

The *EVO ICL V4c* (STAAR Surgical, Monrovia, CA, USA) with a central KS-Aquaport was implanted in the posterior chamber. Lens sizing was determined using **white-to-white (WTW) measurements obtained via the Zeiss IOLMaster (Carl Zeiss Meditec, Germany)**, combined with anterior chamber depth (ACD) data. These biometric parameters guided lens selection to ensure appropriate vaulting and minimize risk of angle closure or lens-crystalline lens contact.

*Postoperative Management and Follow-up* Patients were prescribed topical prednisolone acetate (1%)( **Econopred®** – *Alcon Laboratories, Fort Worth, TX, USA)*, gatifloxacin (0.3%)**( Gatistar®** – *Orchidia Pharmaceutical Industries, Cairo, Egypt)*, and combined tobramycin and dexamethasone phosphate (0.1%) (Tobradex®, Alcon Laboratories Inc., Fort Worth, TX, USA). Follow-up evaluations were conducted 1 day, 1 week, 1 month, 6 months, and 1 year postoperatively.

## Results

### Demographic and baseline characteristics

The study included 117 eyes of 60 patients with moderate-to-high myopia divided into two groups: Group I (Veriflex, 58 eyes) and Group II (ICL, 59 eyes). Both groups were statistically comparable in terms of age, sex distribution, refractive error, and preoperative endothelial cell density (ECD), ensuring a valid comparative analysis (Table 1).

**Table 1:** Demographic and Preoperative Clinical Characteristics.

Characteristic	Group I (Veriflex)	Group II (ICL)	*P*-value
Age (years)	26.3 ± 6.1	27.7 ± 4.1	0.315
Sex (Male:Female )	26:32 (44.8%:55.2%)	27:32 (45.8%:54.2%)	0.865
Refractive Error (SE, D)	−8.5 ± 2.5	−8.2 ± 2.3	0.780
Preoperative ECD (cells/mm^2^)	2683.4 ± 340.6	2732 ± 322.8	0.718

### Endothelial cell density (ECD) changes

**Fig. 1:**
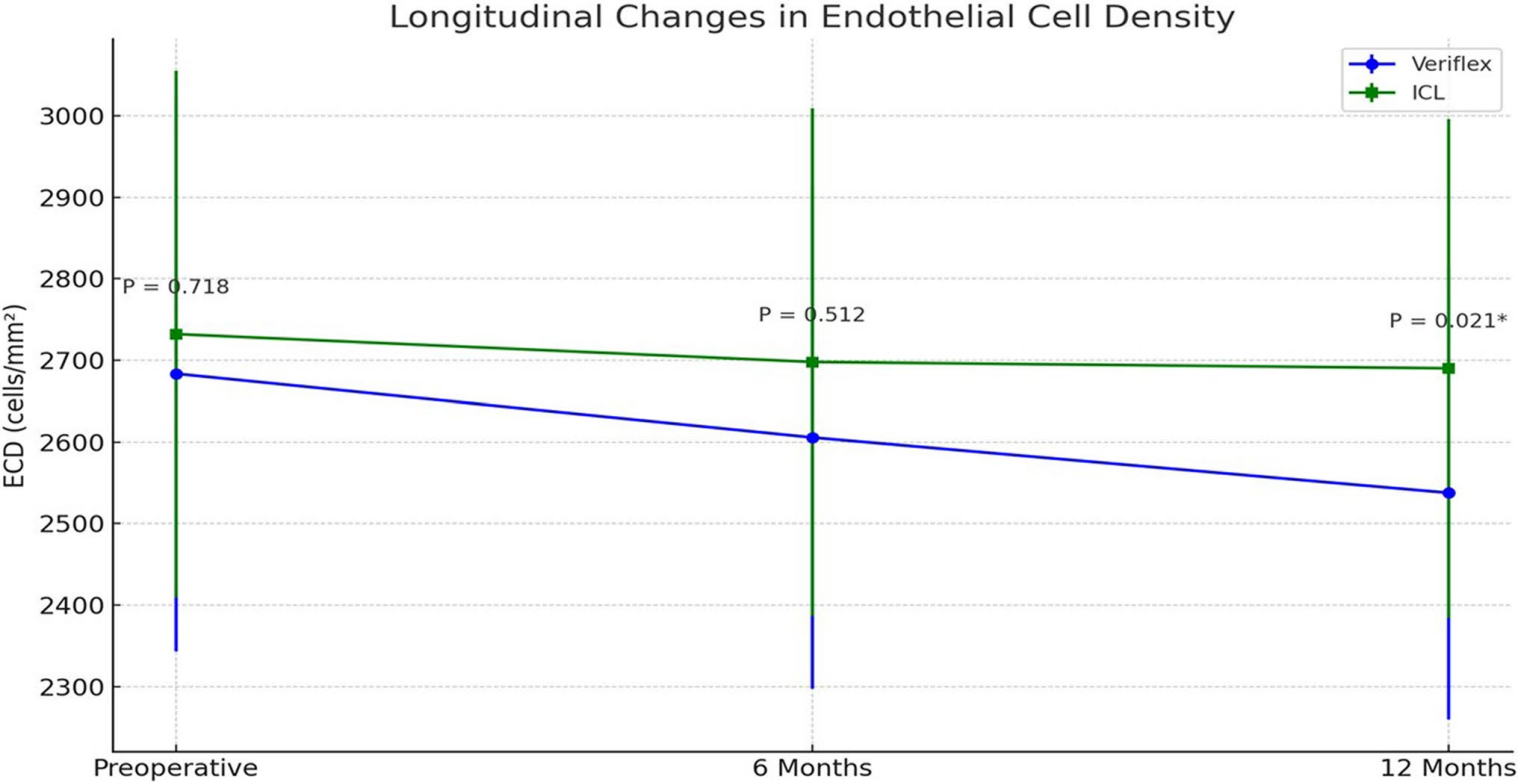
line graph illustrating the longitudinal changes in ECD for both the Veriflex and ICL groups.

Both groups exhibited significant reductions in ECD over the 12-month follow-up period. However, the **magnitude of endothelial cell loss** differed markedly between the groups (Table 2) and Fig. 1

**Table 2:** Longitudinal ECD Changes Over 12 Months.

Timepoint	Group I (Veriflex)	Group II (ICL)	P-value (Between Groups)
Preoperative	2683.4 ± 340.6	2732 ± 322.8	0.718
6 Months	2605.6 ± 308.6	2697.8 ± 311.2	0.512
12 Months	2537.7 ± 277.8	2690.0 ± 305.5	0.021*
Change from Baseline (cells/mm^2^) ± SD	−145.7 ± 62.8	−42.0 ± 17.3	
Percentage Change	−5.4%	−1.53%	
P-value (Within Group)	< 0.001	< 0.001

*Within-Group Analysis:*

- *Veriflex Group* ECD decreased by **145.7 ± 62.8 cells/mm**^**2**^ (5.4%, *p* < *0.001*) at 12 months.
- I*CL Group* ECD decreased by **42 ± 17.3 cells/mm**^**2**^ (1.53%, *p* < *0.001*) at 12 months.

*Between-Group Analysis*

At 6 months postoperatively, Group I (Veriflex) showed a decrease in mean ECD to **2605.6 ± 308.6 cells/mm**^**2**^, while Group II (ICL) showed a mean of **2697.8 ± 311.2 cells/mm**^**2**^. The intergroup difference at this stage was **not statistically significant (*P* = 0.512)**.

At 12 months, the Veriflex group had **significantly lower ECD** compared to the ICL group (*p* = *0.021*), confirming a **3.5-fold greater cell loss** in Veriflex (5.4% vs. 1.53%).

### Corneal biomechanical changes

Corneal biomechanics, assessed via Corvis ST, revealed significant differences between the groups at 12 months (Table 3).

**Table 3:** Corvis ST Parameters at Baseline and 12 Months Postoperatively.

Parameter	Group I (Veriflex)	Group II (ICL)	P-value (Between Groups at 12 Months)	Within-Group Change (*p*-value)
*Deformation Amplitude (DA, mm)*				
- Baseline	1.10 ± 0.12	1.08 ± 0.10	–	–
- 12 Months	1.15 ± 0.11	1.09 ± 0.09	0.032*	Veriflex: *p* = *0.008*
*Highest Concavity Time (HCT, ms)*				
- Baseline	16.8 ± 1.2	16.5 ± 1.1	–	–
- 12 Months	17.2 ± 1.1	16.6 ± 1.0	0.045*	Veriflex: *p* = *0.012*
*Corvis Biomechanical Index (CBI)*				
- Baseline	0.25 ± 0.10	0.24 ± 0.09	–	–
- 12 Months	0.30 ± 0.12	0.25 ± 0.10	0.038*	Veriflex: *p* = *0.006*

*Deformation Amplitude (DA)*

Veriflex corneas became significantly more deformable (DA increased from 1.10 ± 0.12 mm to 1.15 ± 0.11 mm, *p* = *0.008*), while ICL corneas remained stable (*p* > *0.05*).

*Highest Concavity Time (HCT)*

Veriflex corneas took longer to recover from deformation (HCT increased from 16.8 ± 1.2 ms to 17.2 ± 1.1 ms, *p* = *0.012*), indicating reduced stiffness.

*Corvis Biomechanical Index (CBI)*

Veriflex corneas showed reduced biomechanical stability (CBI increased from 0.25 ± 0.10 to 0.30 ± 0.12, *p* = *0.006*), while ICL corneas maintained stable values (*p* > *0.05*).

### Visual outcomes

**Fig. 2:**
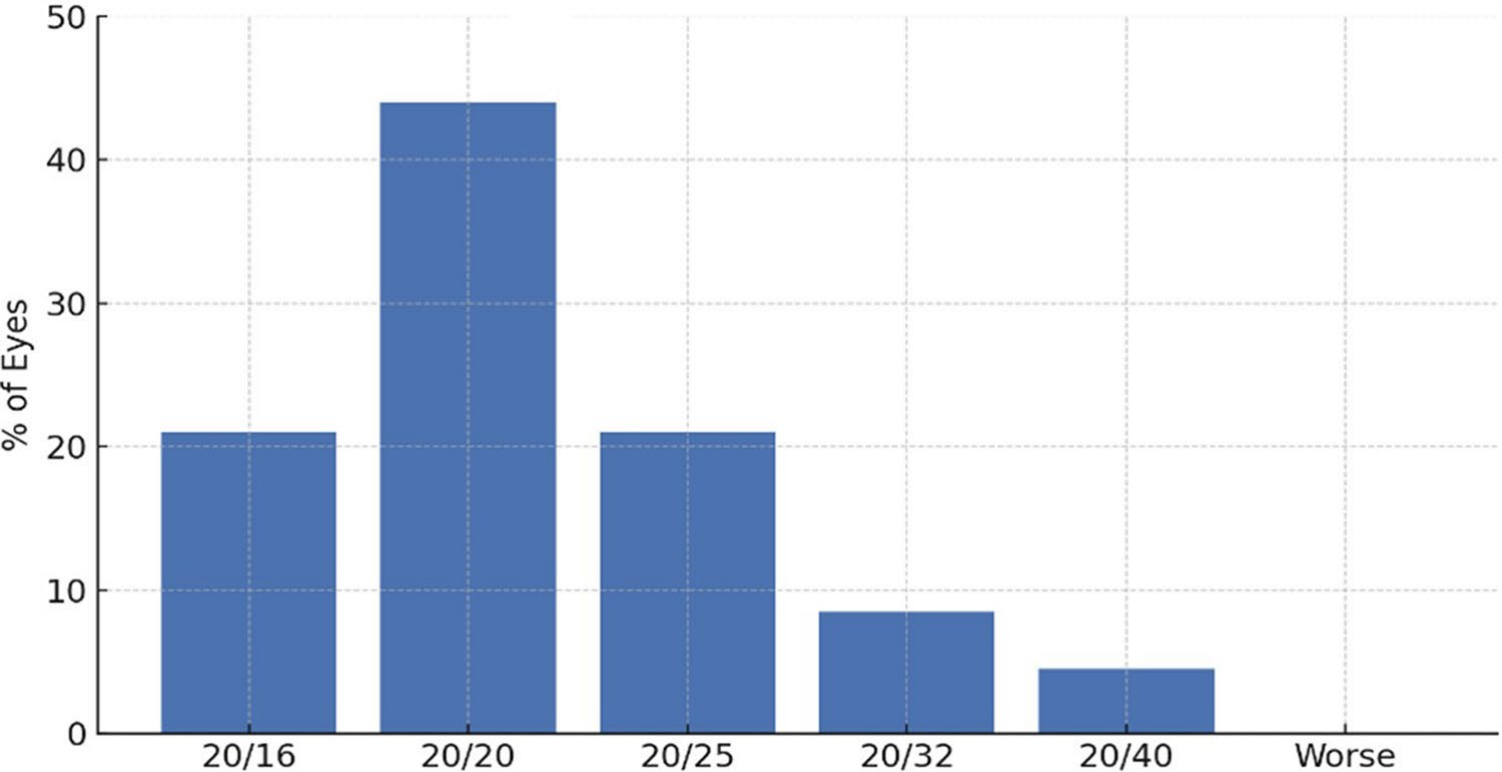
Efficacy: Cumulative uncorrected distance visual acuity (UDVA) at 12 months.

**Fig. 3:**
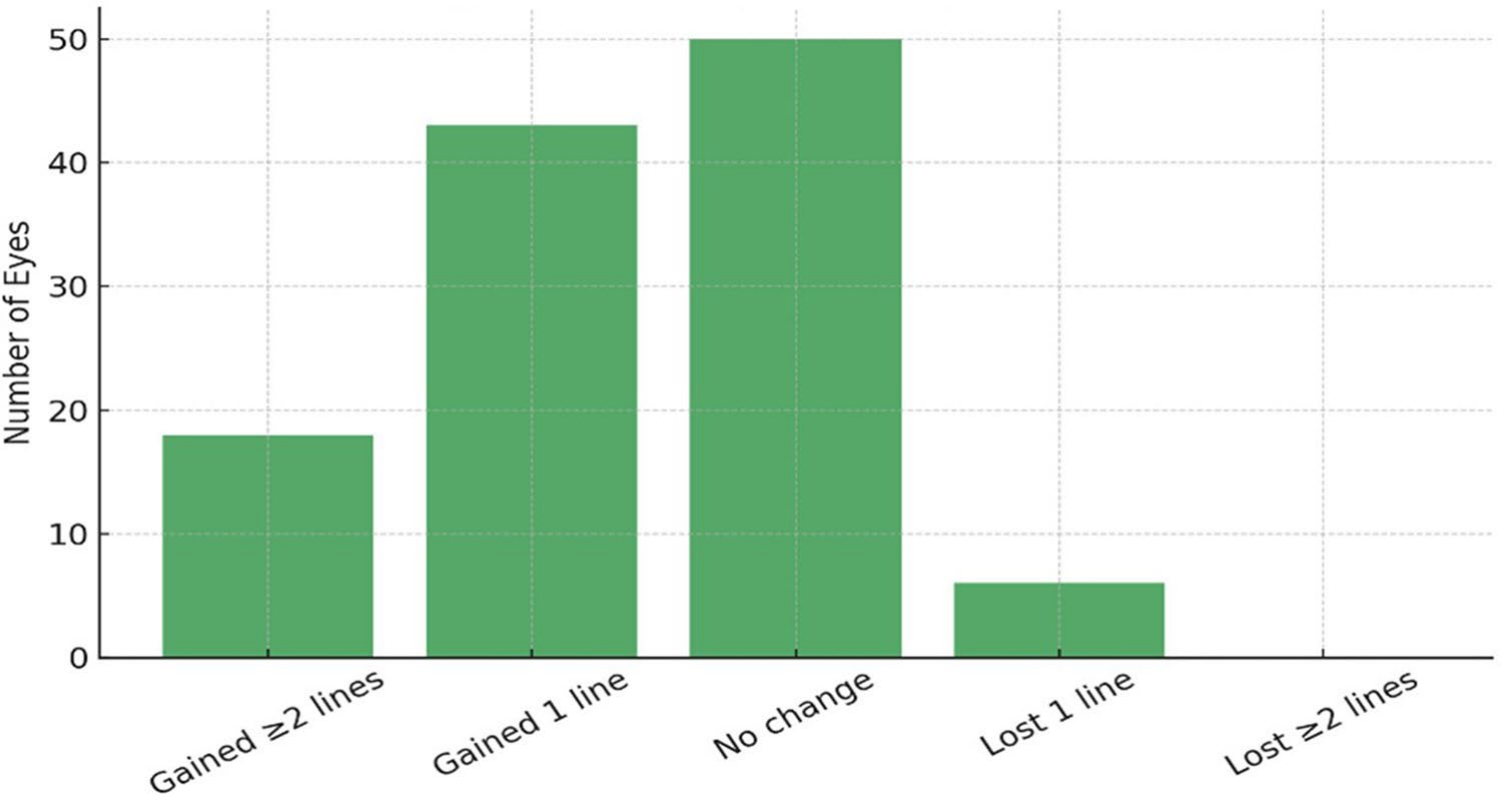
Safety: Change in best-corrected visual acuity (BCVA) from baseline to 12 months.

**Fig. 4:**
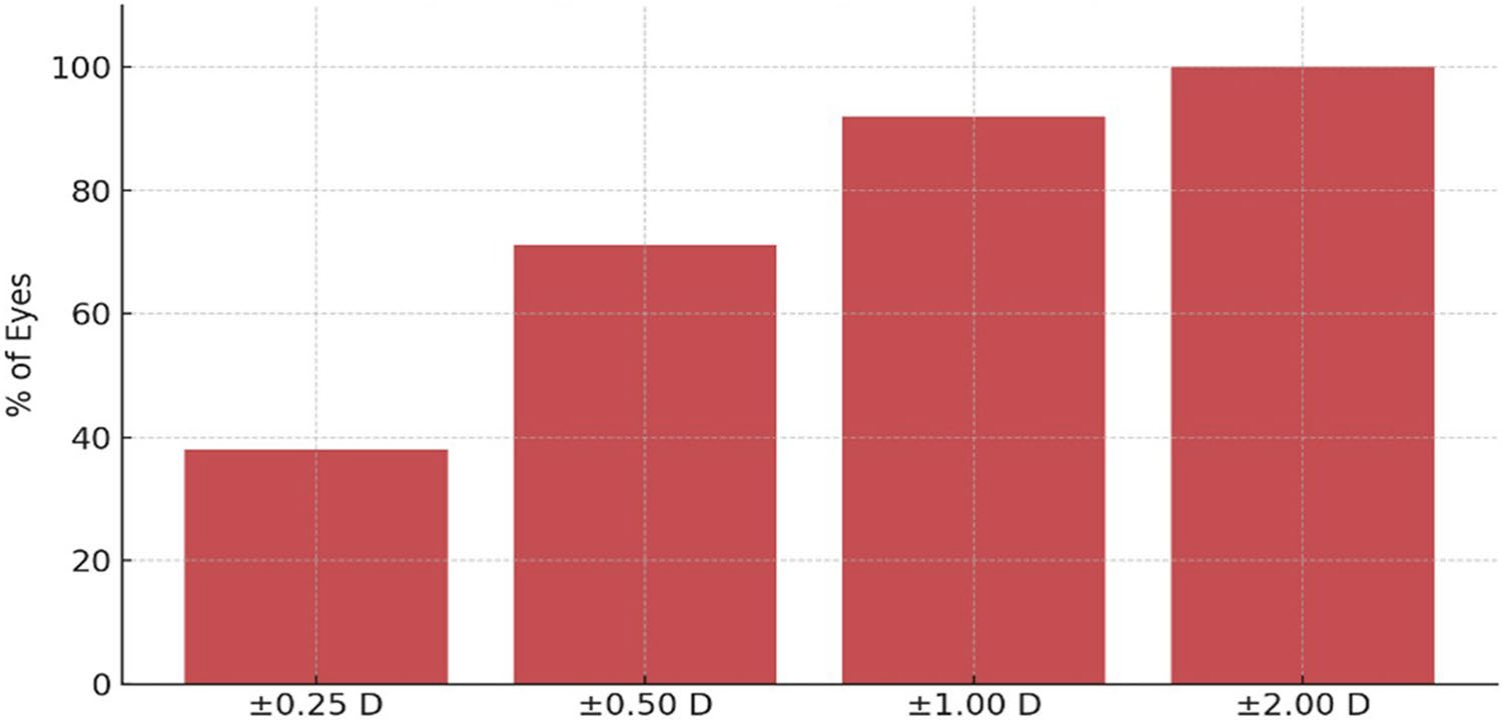
Predictability (Accuracy): Percentage of eyes within ± 0.25 D, ± 0.50 D, ± 1.00 D, and ± 2.00 D of target refraction.

**Fig. 5:**
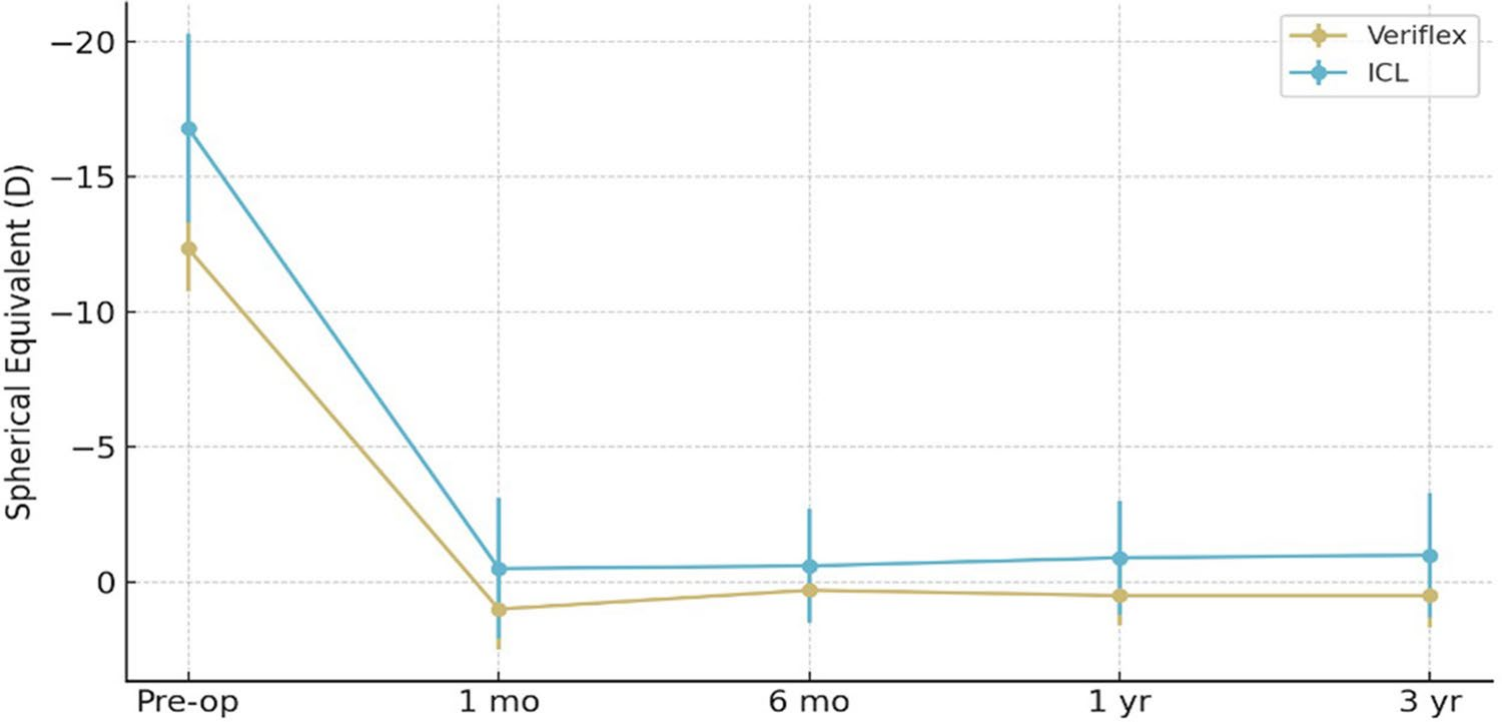
Stability: Mean spherical equivalent (SE) change over time with standard deviation bars.

Both groups achieved comparable improvements in uncorrected visual acuity (UCVA) and best-corrected visual acuity (BCVA) at 12 months (*p* > *0.05*) as shown in Table 4 and Figs. 2, 3, 4 and 5. No intraoperative complications, such as lens dislocation or pupillary block, were reported in either group.

**Table 4:** Visual Outcomes at 12 Months.

Parameter	Veriflex (n = 58 eyes)	ICL (n = 59 eyes)	*p*-value	Interpretation
*Uncorrected Distance Visual Acuity (UDVA)*				
UDVA ≥ 20/25 (%)	83%	89%	0.241	No significant difference
UDVA ≥ 20/20 (%)	39%	49%	0.218	Slight trend favoring ICL
*Best-Corrected Visual Acuity (BCVA)*				
Gained ≥ 2 lines (%)	14%	18%	0.532	No significant difference
Lost 1 line (%)	5.2%	2.5%	0.413	Not significant
*Predictability (Refractive Accuracy)*				
SE within ± 0.50 D (%)	66%	76%	0.207	Trend favoring ICL
SE within ± 1.00 D (%)	89%	95%	0.176	High predictability in both groups
*Stability (SE Change over 12 months)*				
SE mean change (6 to 12 mo)	+ 0.2 ± 0.3 D	−0.3 ± 0.2 D	< 0.001 ★	Significant hyperopic shift in Veriflex
*Endothelial Cell Count (ECC)*				
ECC loss at 12 months (%)	−5.43%	−1.54%	0.021 ★	Significantly more ECC loss in Veriflex

★indicates statistically significant difference at *p* < 0.05.

All data are from the 12-month follow-up interval.

Refractive error (SE) is in diopters (D), mean ± SD.

*Key Findings*

1. Endothelial Safety

- Veriflex caused **clinically significant ECD loss** (5.4%), exceeding the natural annual attrition rate (**0.3–0.6%**)^13^.
- ICL demonstrated **minimal ECD loss** (1.53%), aligning with physiological turnover^6,13,15^.

2. Biomechanical Impact

- Veriflex altered corneal biomechanics, increasing deformability (DA) and reducing recovery speed (HCT), suggesting potential long-term risks for corneal ectasia.
- ICL preserved corneal biomechanical stability, with no significant changes in DA, HCT, or CBI.

3. Clinical Implications

- ICL is superior for preserving endothelial integrity and corneal biomechanics in patients with moderate to high myopia.
- Veriflex should be reserved for cases where posterior chamber implantation is contraindicated, with rigorous postoperative monitoring.
- Mean central vault in the ICL group at 1 month was 525 ± 110 μm (range: 350–790 μm), remaining stable through 12 months.

## Discussion

The increasing global prevalence of myopia, particularly high myopia, necessitates advancements in refractive surgery to ensure long-term safety and efficacy^1^. Phakic intraocular lenses (pIOLs), including iris-fixated anterior chamber lenses (e.g., Veriflex) and posterior chamber implantable Collamer lenses (ICL), are critical alternatives for patients unsuitable for laser-based procedures^2,4,12^. This study’s findings align with recent literature but also highlight critical distinctions between lens types, particularly in endothelial cell density (ECD) preservation and corneal biomechanical stability.

### Endothelial safety: veriflex vs. ICL

The **5.4% ECD loss** observed with Veriflex at 12 months corroborates prior studies reporting significant endothelial cell attrition with anterior chamber pIOLs. Guber et al^6^ demonstrated a 4.8% annual ECD loss with iris-fixated lenses, attributing this to mechanical iris-lens contact and chronic inflammation. Similarly, Yaşa and Ağca^10^ found a 5.2% loss with Veriflex at 5 years, suggesting progressive endothelial trauma. In contrast, the ICL group’s **1.53% loss** aligns with the 1.2–1.8% annual physiological attrition rate^13,14^ and mirrors long-term ICL studies^15,16^.

The ICL’s posterior chamber design minimizes endothelial interaction, reducing mechanical stress ^17^. This is further supported by Amer et al.^8^, who reported stable anterior chamber depth and endothelial parameters after ICL implantation. Conversely, Veriflex’s anterior placement risks intermittent endothelial touch during accommodation, particularly in shallow anterior chambers (< 3.2 mm)^10,18,25,29^.

### Corneal biomechanics: implications for ectasia risk

Corvis ST parameters revealed **increased corneal deformability** (DA: + 0.05 mm, *p* = *0.008*) and **reduced biomechanical stability** (CBI: + 0.05, *p* = *0.006*) in the Veriflex group. These changes mirror findings in keratoconic corneas^19^ and suggest a heightened risk of ectasia, particularly in predisposed patients^20^. The ICL group’s stable biomechanics (DA: + 0.01 mm, *p* > *0.05*; CBI: + 0.01, *p* > *0.05*) aligns with studies showing minimal biomechanical disruption with posterior chamber pIOLs^20,21^.

Anterior chamber pIOLs may alter corneal hysteresis by redistributing intraocular pressure (IOP)-related stress^23^. Recent work by Guo et al.^11^ demonstrated that corneal stiffness increases with age, suggesting that the additional biomechanical changes induced by Veriflex may counteract this natural stiffening, potentially compromising corneal stability in younger patients. In contrast, ICL’s vaulted design preserves corneal structural integrity, as validated by finite element modeling^6,21^.

The observed biomechanical alterations in the Veriflex group—namely increased deformation amplitude and elevated CBI—may be partially attributed to the **larger corneal incision required for Veriflex implantation**. Unlike ICL insertion, which is typically performed through a **2.8–3.0 mm corneal incision**, Veriflex lenses require a **3.2–6.0 mm incision** due to their rigid PMMA material^14,29^. Larger incisions have been shown to induce **altered corneal tensile strength and wound healing dynamics**, particularly in the superior meridian, where incisions are often placed ^24^. Additionally, the **iris-claw fixation** of Veriflex involves mechanical enclavation to the mid-peripheral iris, which may exert **localized tractional forces** on adjacent corneal tissue and anterior segment structures^30,31^. In contrast, the ICL resides entirely within the **posterior chamber**, supported by the ciliary sulcus, and avoids direct mechanical contact with the iris or cornea, thereby preserving **anterior segment integrity and biomechanical stability**^6,21^. These anatomical and procedural distinctions likely contribute to the more pronounced corneal biomechanical shifts observed in Veriflex-implanted eyes despite similar refractive correction.

### Clinical implications and patient selection

The ICL’s superior safety profile supports its preference for most patients, particularly those with borderline endothelial counts (< 2500 cells/mm^2^) or biomechanical vulnerabilities (e.g., forme fruste keratoconus ^19,25^. Recent advancements, such as the central hole ICL (*EVO ICL V4c* ), further reduce cataract risk and improve aqueous humor dynamics^16,26–28^.

All ICL vaults remained within the acceptable safety range (250–750 μm), supporting the observed stability in endothelial cell density and biomechanical parameters.

Veriflex remains viable for patients with contraindications to posterior chamber implantation (e.g., narrow angles, posterior synechiae) but mandates rigorous monitoring. Long-term data from Bohac et al.^29^ and Stulting et al.^30^ emphasize cumulative endothelial risks, with 20-year models predicting critical ECD thresholds (< 1000 cells/mm^2^) in 15–20% of Veriflex patients.

### Limitations and future directions

While this study’s 1-year follow-up captures early postoperative changes, Long-term outcomes (> 5 years) remain critical, as endothelial cell loss may progress with time, while age-related increases in corneal stiffness could modify the biomechanical response to phakic IOLs.^11^. Additionally, the cohort’s mean age (26–28 years) limits generalizability to older populations, where lens-cataract interactions are more relevant^5^. Future studies should integrate Scheimpflug tomography (e.g., Pentacam) with biomechanical assessments^8^ to refine ectasia risk stratification.

## Conclusion

In the evolving landscape of refractive surgery, ICL demonstrates clear advantages in endothelial preservation and biomechanical stability, aligning with global trends favoring posterior chamber pIOLs^3,4^. Veriflex, while effective, necessitates cautious patient selection and lifelong monitoring. These findings underscore the importance of personalized preoperative planning to optimize visual outcomes and mitigate long-term risks in myopia correction.

## Data Availability

The datasets used and/or analysed during the current study available from the corresponding author on reasonable request.
